# Chiropractic conservatism among chiropractic students in Denmark: prevalence and consequences

**DOI:** 10.1186/s12998-020-00352-3

**Published:** 2020-12-04

**Authors:** Casper Glissmann Nim, Henrik Hein Lauridsen, Søren O’Neill, Guillaume Goncalves, Rikke K. Jensen, Charlotte Leboeuf-Yde

**Affiliations:** 1 Medical Research Unit, Spine Centre of Southern Denmark, University Hospital of Southern Denmark, Østre Hougvej 55, 5500 Middelfart, Denmark; 2 Department of Regional Health Research, University of Southern Denmark, Winsløwsparken 19, 5230 Odense M, Denmark; 3 Department of Sports Science and Clinical Biomechanics, University of Southern Denmark, Campusvej 55, 5230 Odense M, Denmark; 4 Institut Franco Européen de Chiropraxie, 24 Boulevard Paul Vaillant Couturier, 94200 Toulouse, France; 5 Nordic Institute of Chiropractic and Clinical Biomechanics, University of Southern Denmark, Campusvej 55, 5230 Odense M, Denmark

**Keywords:** Chiropractic students, Chiropractic conservatism, Spinal subluxation, Spinal adjustments, Contra-indication, Non-indication, Indication, Education, Survey

## Abstract

**Background:**

The chiropractic profession is split between those practicing evidence-based and those whose practice is honed by vitalism. The latter has been coined ‘chiropractic conservatism’. In Denmark, the chiropractic education program is university-based in close collaboration with a medical faculty. We wanted to investigate if such conservative attitudes were present in this environment.

Our objectives were to i) determine the level of chiropractic conservatism, ii) investigate if this was linked to academic year of study, iii) determine the level of clinical appropriateness, and iv) to investigate if this was affected by the level of conservatism among students in a chiropractic program, where the students are taught alongside medical students at the University of Southern Denmark (SDU).

**Methods:**

A cross-sectional survey of 146 (response-rate 76%) 3rd to 5th year pre-graduate students and 1st year postgraduate clinical interns from the chiropractic degree course at the University of Southern Denmark was conducted during autumn of 2019. The students’ levels of conservatism were dichotomized into appropriate/inappropriate, summed up, and used in a linear regression model to determine the association with academic year of study. Thereafter, the conservatism score was categorized into four groups (from low -1- to high -4-). Conservatism groups were cross-tabulated with the ability to answer appropriately on nine cases concerning i) contra-indications, ii) non-indications, and iii) indications for spinal manipulation and analyzed using logistic regression.

**Results:**

Generally, the Danish chiropractic students had low conservatism scores, decreasing with increasing academic year of study. Seventy percent of the students were placed in the two lowest conservative groups. The level of conservatism (categories 1–3) was moderately (but not statistically significantly) associated with an inability to recognize non-indications to treatment. Three outliers (category 4), however, revealed a highly inappropriate handling of the clinical cases.

**Conclusions:**

Chiropractic students enrolled at a university-based course closely integrated with a medical teaching environment are not immune to chiropractic conservatism. However, the course appears to attenuate it and limit its effect on clinical decision-making compared to other educational institutions.

**Supplementary Information:**

The online version contains supplementary material available at 10.1186/s12998-020-00352-3.

## Background

Two factions exist in the chiropractic profession, which disagree on basic but central principles [1]. Somewhat oversimplified, one group is progressive and evidence-based regarding the chiropractic scope of practice as primarily related to musculoskeletal disorders. The other group considers manual treatment of the spine (denoted ‘*adjustments’)* i.e. *spinal manipulation* as a panacea, purported to effect positive or curative changes in a multitude of diseases, irrespective of the underlying etiology. They can be said to adhere to chiropractic conservatism [1]. Here, we define ‘conservatism’ as acceptance of traditional chiropractic concepts as the Oxford dictionary defines the term conservatism as “a commitment to traditional values and ideas with opposition to change or innovation” [2]. Throughout the article, chiropractic conservatism is used to reflect adherence to traditional and philosophical chiropractic values.

The theoretical basis for choosing a conservatism approach has taken slightly different guises over time. This includes vitalistic concepts of ‘*life-forces*’ with theological undertones [3] to scientific-sounding but fuzzy notions about perturbations of autonomic nervous activity affecting specific organs [4] or, more recently, that ‘adjustments’ have a positive effect on clusters of dormant neurons in the brain [5]. Common to these theories is the conviction that the human body can maintain optimal health if the nervous system is allowed to regulate all tissues without interference. *Furthermore, mechanical spinal dysfunctions (denoted ‘subluxations’) are thought to cause such interferences and are purportedly amenable to spinal ‘adjustments’.* This is the central concept in the traditional chiropractic style. By the same line of thinking, chiropractic treatment is purported also to boost the immune system, and, therefore, some chiropractors are even vocal opponents of vaccination programs [6, 7].

It is disturbing how conservative concepts that lack general contemporary acceptance in the scientific community remain among some chiropractors. Remarkably, these unscientific concepts also find fertile ground in modern-day chiropractic students [8, 9], which is especially troubling. Notably, the degree of chiropractic conservatism restricts the students’ clinical sense of appropriateness, as it is associated with the inability to limit chiropractic practice to indicated cases [9].

However, the chiropractic educational system differs considerably across the globe. We speculate that there are three main types of chiropractic ‘educational patterns’ with different levels of tolerance to chiropractic conservatism. These are:

1. Private and independent chiropractic schools that a) might partially or wholly accept and encourage some degree of conservative and vitalistic approach to chiropractic, or b) do not have the resources to adequately address and deal with such concepts.
2. State university-affiliated, but otherwise independent chiropractic degree courses that are not integrated closely with other healthcare educations. Possibly, they retain teaching staff who, more or less overtly, include elements of chiropractic conservatism in their interactions with students.
3. University chiropractic degree courses that are closely integrated with a medical faculty. The Danish chiropractic course is such a program. Namely, a university-grounded five-year chiropractic degree (Clinical Biomechanics) at the University of Southern Denmark (Odense, Denmark), followed by a one-year clinical internship. The program is placed within a lively research environment, where the members of the research unit, to a large extent, plan the content of the profession- and academic-related subjects, which includes their research in the teachings. Classes related to biomedicine are taught by academic specialists (anatomists teach anatomy, pathologists teach pathology, etc.) as opposed to chiropractors with a *special interest*, and there is considerable overlap in the curriculum with the medical degree course. In fact, chiropractic and medical students follow the same classes and attend the same examinations throughout the 3 years of bachelor studies and, to some degree, in the master’s studies. Pre-graduate clinical training occurs primarily in an outpatient Spine Centre at a publicly funded hospital, where the students attend to patients on referral from general medical practitioners, private practice chiropractors, and other hospital departments. Students are supervised by medical specialists as well as senior chiropractors [10–12].

While no study has looked at chiropractic students in such an environment, chiropractic students from a private and independent institution in Europe responded to a questionnaire on chiropractic conservatism, which revealed that a majority of students held very conservative views on the nature of chiropractic [9]. Further, a robust positive association was reported between a firm adherence to the chiropractic conservative belief system and the willingness to treat non-indicated cases, although, they seemed to have a reasonable approach to indications and contra-indications. Another survey with students attending two chiropractic courses provided at Australian state universities but not closely related with any medical faculty provided information on conservatism and clinical appropriateness but unfortunately did not link them together [8]. There, many chiropractic students expressed very conservative views on spinal ‘adjustments’ and, in general, only minor improvements or even worsening were observed with increasing year of study [8]. As with the private college students, only around 50–60% answered appropriately for non-indicated cases to spinal manipulation. However, also most of these students responded on items relating to contra-indications and indications in an appropriate manner [13].

Of the healthcare professions, allopathic medicine has the longest and most well-established tradition for scientific inquiry [14]. Thus, such outdated concepts should not easily establish themselves without ridicule in a chiropractic program such as the one in Denmark with close co-operation with a medical faculty.

### Aims

The objectives of this study were to i) establish the level of conservatism in chiropractic students at the University of Southern Denmark, ii) investigate if this was linked to academic year of study, iii) determine the level of clinical appropriateness for spinal manipulation in terms of contra-indications, non-indications, and indications, and iv) investigate if this ability was related to the level of conservatism, after controlling for sex and year of study.

We hypothesized that there would be almost no conservative students at this university course, that their ability to identify indications and contra-indications would be similar to other schools [9, 13], but that they would be better at observing non-indications to spinal manipulation.

## Method

### Settings

A cross-sectional, anonymous, and voluntary survey was distributed to 3rd to 5th year students attending the chiropractic program at the University of Southern Denmark, and to recent graduates enrolled in the obligatory 1-year internship program in either a primary or secondary care practice in Denmark.

### Survey development

#### Translation

The full questionnaire has been used previously in a study on chiropractic students from a European private chiropractic institution [9], and a subset of items has been used in a study conducted in two Australian state university schools not collaborating with a medical faculty [8, 13].

The questionnaire was translated from English to Danish using a modified version of Beaton’s cross-cultural adaptation technique [15]. Two staffers from the research team, fluent in English and Danish, translated the survey forward (SON) and backward (HHL). A consensus meeting, where content issues were discussed, was held between SON, HHL, and CGN, and the final Danish version was agreed upon. Subsequently, we pilot-tested the survey on four recently graduated Danish chiropractors, who were interviewed about their understanding of the phrasing and the appropriateness of the items. This did not give rise to any changes to the survey. It took approximately 10 min to complete the survey, as noted during the pilot process. The survey instrument consisted of two parts, and the complete questionnaire is provided in Supplementary File 1.

#### Conservatism

The survey included a questionnaire with ten statements regarding beliefs about spinal ‘adjustment’/manipulation (*n* = 6) and spinal ‘subluxations’/dysfunctions (*n* = 4). These statements were designed to investigate the level of chiropractic conservatism, i.e. the degree to which the respondent agreed with historical, dogmatic ideas about chiropractic, using a five-point Likert scale anchored from *‘Definitely not’/‘Strongly disagree’ (*score = 0) to *‘Yes, definitely’/‘Strongly agree’* (score = 4)*.*

### Clinical appropriateness

The survey further included 9 clinical cases concerning I) low back pain (*n* = 4), II) neck pain (*n* = 3), and III) primary prevention in a child (*n* = 2). These three sets of clinical cases were used to assess students’ ability to detect contra-indications, non-indications, and indications for spinal manipulation (See Table 1).

**Table 1:** Definitions of the different indications for treatment used in a survey on chiropractic students attending the University of Southern Denmark

**Contra-indication:** Cases where spinal manipulation would be associated with a non-trivial risk of complications e.g. manipulation of spinal fracture. **Non-indication:** Cases where spinal manipulation is not contra-indicated, but where no evidence-based clinical rationale is present for offering the treatment e.g. manipulation offered as treatment of asthma or as prevention for infection. **Indication:** Cases where spinal manipulation is not contra-indicated, and musculoskeletal symptoms are present with clinical findings providing an evidence-based rationale for spinal manipulation e.g. non-specific spinal pain	

The cases were presented as an introductory ‘baseline’ vignette, followed by a number of different potential developments over time. Participants were asked to consider clinical options for each of the potential developments.

#### Low back pain questionnaire

A subset of four items had been adopted from a previously published and validated questionnaire [16–18] relating to a case of a 40-year old male with local LBP and no additional musculoskeletal complaints. The case is described in four different ways: i) no prior LBP and complete remission after two sessions, ii) previous recurrent LBP and complete remission after 2 weeks, iii) previously 1 year of intermittent LBP and gradual worsening over six sessions, iv) previously 1 year of intermittent LBP, minor (clinically irrelevant) improvement after six sessions and possible undiagnosed underlying depression.

The respondents were asked to choose one of seven possible strategies for each of the four case-developments a) second opinion, b) additional treatment, c) ‘quick-fix’, d) try again, e) symptom guided maintenance care (patient-administered), f) clinical guided maintenance care (clinician-administered), and finally, students could reply g) *other* and add an answer in the comment section. Case i) was a considered a ‘quick fix’, case ii) a maintenance care patient and cases iii) and iv) were considered to be non-indicated for continued care because iii) was a case where the patient does not respond to spinal manipulation but gradually worsens and iv) indicates a non-mechanical LBP pattern most likely due to a non-musculoskeletal condition (depression).

#### Neck pain questionnaire

A subset of three items was adopted from a questionnaire previously used in a study of French chiropractors [19]. The case describes a 28-year old male tennis player with neck pain and antalgic head position. The case develops in the following three ways: i) Simple mechanical, local neck pain, ii) simple mechanical neck pain with radiation to the trapezius muscle, and iii) development of signs of an upper motor lesion. The first two cases were considered indications to treatment, whereas the third was considered an obvious contra-indication.

#### Primary prevention in a child

We included two additional items from the previous study of the private college [9] regarding primary prevention. The first case concerns the mother of a 5-year old child with no prior spinal pain, who consults a chiropractor, asking if the chiropractor would be able to treat the child prophylactically to avoid future spinal pain. The second case describes the mother of a 5-year old child with a long family history of multiple conditions, breast cancer, diabetes etc., who asks if the chiropractor would be able to treat the child prophylactically to avoid the onset of diseases in the future. Both cases were obvious non-indications to spinal manipulation.

#### Interpretation of “other”

The students also had the option to answer *other* and write a comment. All such comments were read thoroughly and independently by two authors (CGN and SON). In cases of agreement or when consensus could be reached, the ‘other’ answers were re-classified into one of the fixed answer possibilities or left under ‘other’. If consensus could not be achieved, a third author (HHL) arbitrated the decision.

### Survey distribution

Information about the project (Supplementary file 2) was distributed beforehand by e-mail on October 1st, 2019, and two reminders followed on the 8th and 22nd of October using the student e-mail system. In order to achieve as high a response rate as possible, a lecturer or one of the researchers (CGN) interrupted the students’ regular classwork to provide the possibility to answer the survey there-and-then, or later as they preferred. Clinical interns were informed about the study during a meeting pre-scheduled as part of the internship program (Oct. 27th, 2019). Time was allowed at the meeting to complete the survey. No reminders were sent. The survey was conducted using SurveyXact (Aarhus, Denmark) [20], and all data were collected anonymously online. No attempts were made to identify any of the respondents based on their demographic data or replies. In Denmark, no ethics permission is necessary to conduct an anonymous questionnaire survey [21].

### Variables of interest

The data in question were extracted directly from the online storage at SurveyXact and downloaded as a comma-separated values file.

#### Chiropractic conservatism

Each response to the 10 items concerning chiropractic conservatism was dichotomized into ‘appropriate’/‘inappropriate’, as described in Supplementary File 1. This definition of ‘appropriate’ and ‘inappropriate’ was the same as the one used in the original study [9], namely that a conservative view was considered ‘inappropriate’ e.g. agreeing that spinal ‘adjustments’ can boost the immune system. The degree of conservatism was calculated as the number of ‘inappropriate’ answers, yielding an individual score between 0 and 10. Thus, the higher the score, the more conservative the student was considered to be. This score was further categorized into four conservatism groups ranging from low to high conservatism: Group 1, a score between 0 and 2; group 2, a score between 3 and 5; group 3, a score of 6 or 7; and finally group 4, a score between 8 and 10, using the same scoring system as in the private college study [9].

#### Clinical appropriateness

Answers to the nine clinical cases were dichotomized into an ‘appropriate’ answer (0) and an ‘inappropriate’ answer [1] as defined in the primary publication [13]. The rationale for these is described in Supplementary File 3.

Self-reported data on academic year of study (3rd to 5th year and postgraduate interns), age, and sex were also collected before commencing the questionnaire survey.

### Statistical analysis

Descriptive data for age is reported as the mean and standard deviation. Sex and year of study are reported as frequencies. The internal consistency for the survey pertaining the conservatism score is evaluated using Cronbach’s Alpha with 95% confidence intervals. An alpha score higher than 0.7 is considered acceptable [22]. The proportion of ‘appropriate’ answers to the clinical items is visualized as bar graphs for each item per academic year, including error bars representing 95% confidence interval.

#### Associations between conservatism and academic year of study

Binary and multivariate linear regression analyses were performed using the conservatism score [0–10] as the dependent variable and the academic year of study as the independent variable. The analysis was then adjusted for sex. Associations are presented as beta coefficients with 95% confidence intervals.

#### Associations between clinical appropriateness and conservatism

The associations between the conservatism group [1–4] and clinical appropriateness were tested for statistical significance using logistic binomial regression, both unadjusted and adjusted for sex and academic year. All associations are presented as odds ratios (OR) with 95% confidence intervals. Non-overlapping of confidence intervals would determine if differences were statistically significant.

Data importing, cleaning, and data analyses were performed in R (Vienna, Austria) version 3.6 with R-studio version 1.3 for Linux [23] using the Tidyverse [24]. The R package *exactci* [25] was used to calculate 95% confidence intervals.

## Results

### Descriptive student data

One hundred and sixty-seven students and 24 interns i.e. recently graduated chiropractic students enrolled in the obligatory 1-year internship program (*N* = 191), were invited to participate in the study, and 146 (response rate 76%) completed the survey. Of these, 80 (55%) were female, and the mean age was 25.7 years (SD = 2.9). Response rates were lowest in the 3rd year and highest for the interns. For further details, see Table 2.

**Table 2:** The response rates for participation in a survey of Danish chiropractic students and postgraduate interns

Academic year of study	Males n (% of students)	Females n (% of students)	% of respondents per academic year of study of all eligible students
*Interns*	10 (100)	14 (100)	100
*5th year*	19 (79)	29 (97)	89
*4th year*	15 (75)	20 (74)	74
*3rd year*	19 (61)	17(57)	59
*Missing responses*	3 (one 3rd year and, two 4th year students)		

### Conservative beliefs

For the conservative beliefs, the Cronbach’s alpha score was 0.84 [0.81–0.88], with no single item ranking below 0.82, indicating that the survey has acceptable internal consistency. Table 3 lists the distribution of answers concerning chiropractic conservatism in absolute numbers, percentages, and 95% confidence intervals by year of study and in total. The ‘inappropriate’ answers per item ranged between < 1 and 68%, with 6 of the 10 items found to be below 33%. The mean and median numbers of ‘inappropriate’ answers per student were both 3/10 .

**Table 3:** Distribution of answers by Danish chiropractic students and postgraduate interns concerning spinal manipulation and adjustments

Items concerning spinal adjustments	Year	N	Definitely not/ Probably not n (%) [95%CI]	Don’t know n (%) [95%CI]	Yes, probably/ Yes, definitely n (%) [95%CI]
*Can spinal adjustments prevent disease in general?*	3	36	21 (58) [40–75]	2 (6) [0–19]	13 (36) [21–54]
	4	37	24 (65) [48–80]	5 (14) [5–29]	8 (22) [10–38]
	5	48	39 (81) [67–91]	5 (10) [3–23]	4 (8) [2–20]
	Interns	24	19 (79) [58–93]	3 (12) [3–32]	2 (8) [1–27]
	Total	145	103 (71) [63–78]	15 (10) [6–16]	27 (19) [13–26]
*Can spinal adjustments help the immune system?*	3	36	21 (58) [41–75]	8 (22) [10–39]	7 (19) [8–36]
	4	36	24 (67) [49–81]	4 (11) [3–26]	8 (22) [10–39]
	5	48	44 (92) [80–98]	3 (6) [1–18]	1 (2) [0–11]
	Interns	24	19 (79) [58–93]	3 (12) [3–32]	2 (8) [1–27]
	Total	144	108 (75) [67–82]	18 (12) [8–19]	18 (12) [8–19]
*Can spinal adjustments improve the health of infants?*	3	36	8 (22) [10–39]	6 (17) [6–33]	22 (61) [43–77]
	4	37	5 (14) [5–29]	6 (16) [6–32]	26 (70) [53–84]
	5	48	14 (29) [17–44]	11 (23) [12–37]	23 (48) [33–63]
	Interns	23	4 (17) [5–37]	6 (25) [10–47]	13 (57) [33–74]
	Total	144	31 (22) [15–29]	29 (20) [14–27]	84 (58) [49–66]
*Can adjustments help the body function at 100% of its capacity?*	3	36	7 (19) [8–36]	2 (6) [0–19]	27 (75) [58–88]
	4	37	9 (24) [12–41]	2 (5) [0–18]	26 (70) [53–84]
	5	48	16 (33) [20–48]	6 (12) [5–25]	26 (54) [39–69]
	Interns	24	4 (17) [5–38]	–	20 (83) [63–95]
	Total	145	36 (25) [18–33]	10 (7) [3–12]	99 (68) [60–76]
*Can spinal adjustments prevent degeneration of the spine?*	3	36	11 (31) [16–48]	8 (22) [10–39]	17 (47) [30–65]
	4	36	17 (47) [30–65]	9 (25) [12–42]	10 (28) [14–45]
	5	48	31 (65) [49–78]	8 (17) [7–30]	9 (19) [9–33]
	Interns	24	16 (67) [45–84]	2 (8) [1–27]	6 (25) [10–47]
	Total	144	75 (52) [44–60]	27 (19) [13–26]	42 (29) [22–37]
*It is appropriate for every person to receive chiropractic adjustments for their entire life?*	3	34	17 (50) [32–68]	6 (18) [7–35]	11 (32) [17–51]
	4	37	18 (49) [32–66]	9 (24) [11–41]	10 (27) [14–44]
	5	48	38 (79) [65–90]	2 (4) [0–14]	8 (17) [7–30]
	Interns	24	19 (79) [58–93]	3 (12) [3–32]	2 (8) [1–27]
	Total	143	92 (64) [56–72]	20 (14) [9–21]	31 (22) [15–29]
**Items concerning spinal subluxation**	**Year**	**N**	**Highly disagree/ disagree** **n (%) [95%CI]**	**Don’t know** **n (%) [95%CI]**	**Highly agree/** **agree** **n (%) [95%CI]**
*Subluxations are the cause of all disease.*	3	34	33 (97) [85–100]	–	1 (3) [0–15]
	4	37	36 (97) [86–100]	1 (3) [0–14]	–
	5	48	47 (98) [89–100]	1 (2) [0–11]	–
	Interns	24	24 (100) [86–100]	–	–
	Total	143	140 (98) [94–100]	2 (1) [0–5]	1 (< 1) [0–4]
*Subluxations cause short-circuits of the nervous system.*	3	33	24 (73) [54–87]	2 (6) [0–20]	7 (21) [9–39]
	4	37	24 (65) [47–80]	5 (14) [5–29]	8 (22) [9–38]
	5	48	44 (92) [80–98]	1 (2) [0–11]	3 (6) [1–17]
	Interns	24	16 (67) [45–84]	3 (12) [3–32]	5 (21) [7–42]
	Total	142	108 (76) [68–83]	11 (8) [4–13]	23 (16) [11–23]
*Subluxations can have a negative effect on the capacity of the nervous system to provide energy to tissues and organs.*	3	33	12 (36) [20–55]	3 (9) [2–24]	18 (55) [36–72]
	4	37	13 (35) [20–52]	9 (24) [11–41]	15 (41) [25–58]
	5	48	40 (83) [70–93]	2 (4) [0–14]	6 (12) [5–25]
	Interns	24	11 (46) [25–67]	3 (12) [3–32]	10 (42) [22–63]
	Total	142	76 (54) [45–62]	17 (12) [7–18]	49 (35) [27–43]
*It is possible to detect subluxations before symptoms appear.*	3	33	9 (27) [13–46]	6 (18) [7–35]	18 (55) [36–72]
	4	36	9 (25) [12–42]	8 (22) [10–39]	19 (53) [35–70]
	5	48	26 (54) [39–69]	10 (21) [10–35]	12 (25) [14–40]
	Interns	24	7 (29) [13–51]	4 (17) [5–37]	13 (54) [33–74]
	Total	141	51 (36) [28–45]	28 (20) [14–27]	62 (44) [36–53]

“Yes, probably”, “Yes, definitely”, “Agree”, and, “Strongly agree” were considered inappropriate answers

In general, the students had more ‘inappropriate’ beliefs about ‘spinal adjustments’/manipulation than about ‘subluxations’/dysfunction. Two items concerning ‘adjustments’/manipulation scored more than 50% of ‘inappropriate’ answers; 68% accepted that *spinal adjustments can help the body function at 100% of its capacity*, and 58% reported that they believe that *spinal adjustments have the ability to improve the health of infants*.

On items regarding ‘subluxations’, 43% believed that it is possible to detect *subluxations before the onset of symptoms*, but only one student believed in the original chiropractic concept that *subluxations are the cause of all diseases*.

The number of ‘inappropriate’ answers per student by year of study is listed in Table 4, and only 3 students scored 8 or more (highly conservative).

**Table 4:** Number of ‘inappropriate’ answers per student in a survey of Danish chiropractic students and postgraduate interns

Number of inappropriate answers per student	3rd year *n* = 37 n (%) [95%CI]	4th year n = 37 n (%) [95%CI]	5th year *n* = 48 n (%) [95%CI]	Postgraduate interns *n* = 24 n (%) [95%CI]
0	4 (11) [3–25]	2 (5) [0–18]	8 (17) [7–30]	2 (8) [1–27]
1	2 (5) [0–18]	4 (11) [3–25]	17 (35) [22–51]	3 (6) [3–32]
2	3 (8) [2–22]	9 (24) [11–41]	9 (19) [9–33]	2 (8) [1–27]
3	4 (11) [3–25]	1 (3) [0–14]	7 (15) [6–28]	6 (21) [10–47]
4	5 (14) [5–29]	7 (19) [8–35]	2 (4) [1–14]	4 (17) [5–37]
5	6 (16) [6–32]	4 (11) [3–25]	3 (6) [13–17]	4 (17) [5–37]
6	3 (8) [2–22]	4 (11) [3–25]	1 (2) [0–11]	1 (4) [0–21]
7	4 (11) [3–25]	2 (5) [0–18]	1 (2) [0–11]	1 (4) [0–21]
8	1 (3) [0–14]	1 (3) [0–14]	–	–
9	1 (3) [0–14]	–	–	–
10	–	–	–	–
Missing data	4 (11) [3–25]	3 (8) [2–22]	–	1 (4) [0–21]

#### Changes in conservatism score by academic year of study

A clear association between the degree of conservatism (0–10) and academic year of study was observed for the Danish students. The conservatism score was found to decrease by each increasing academic year at the university, reaching statistical significance at the 5th year, also after adjusting for sex. However, there was no statistically significant difference between 3rd year students and postgraduate interns (Table 5).

**Table 5:** Association between chiropractic conservatism group and year of study in a survey of Danish chiropractic students and postgraduate interns (n = 146)

Comparisons between academic years	β Estimate [95% CI] Unadjusted	β Estimate [95% CI] adjusted for sex [index = female]
*Year 3 compared to year 4*	−0.5 [−1.5–0.5]	− 0.5 [− 1.5–0.5]
*Year 3 compared to year 5*	**−2.1 [−3.0 – − 1.2]**	**−2.2 [− 3.1 – − 1.3]**
*Year 3 compared to Interns*	−0.9 [− 2.0–0.2]	−1.0 [− 2.1–0.1]

#### Sum of conservatism scores collapsed into four categories

When the total number of ‘inappropriate’ answers per student was categorized into four groups by level of conservatism, 66 (45%) were placed in conservative group 1, 52 (36%) in conservative group 2, 17 (12%) in group 3, and only 3 (2%) participants belonged to the extremely conservative group 4, i.e. most students were in the least conservative groups (1 and 2). Eight participants (5%) failed to complete one or more items and could not be categorized. They reported a mean conservative score of 2.7 (0–10) and had an average of 3.7 unanswered items out of the 10 possible items.

### Clinical appropriateness

For the clinical cases, a total of 72 answers were provided in the ‘other’ category, which allowed for written comments. After carefully reviewing these, 24 were re-categorized as the comment was already covered by one of the provided ‘fixed’ answer options. This resulted in 2.1% of all answers being re-categorized from ‘other’, which by definition was scored as ‘inappropriate’ to an ‘appropriate’ fixed answer, i.e. an appropriate clinical rationale.

#### Ability to determine contra-indications

**Fig. 1:**
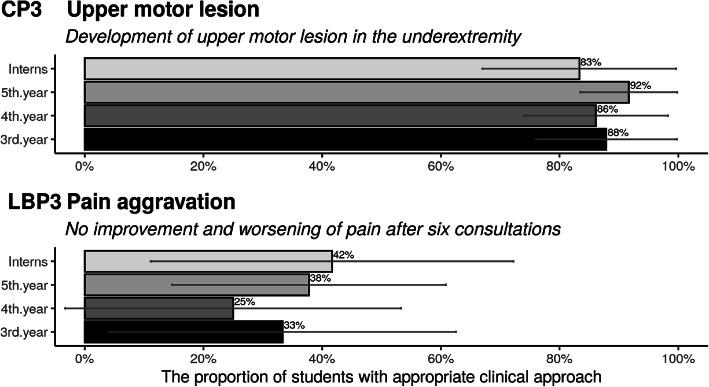
Proportions of Danish chiropractic students and postgraduate interns who were able to select contra-indications for spinal manipulation. (Error bars represent 95% confidence intervals (*n* = 146))

Participants’ ability to recognize contra-indications, grouped by academic year of study, is presented in Fig. 1. For the first clinical case (patient with an upper motor lesion), most students, across classes, correctly recognized the need for referral (between 83 and 92%). By contrast, for the clinical case with the patient who gradually got worse with treatment, only between 25 and 42% would refer this patient for a second opinion. However, the confidence intervals were substantial for the second case.

#### Ability to determine non-indications

**Fig. 2:**
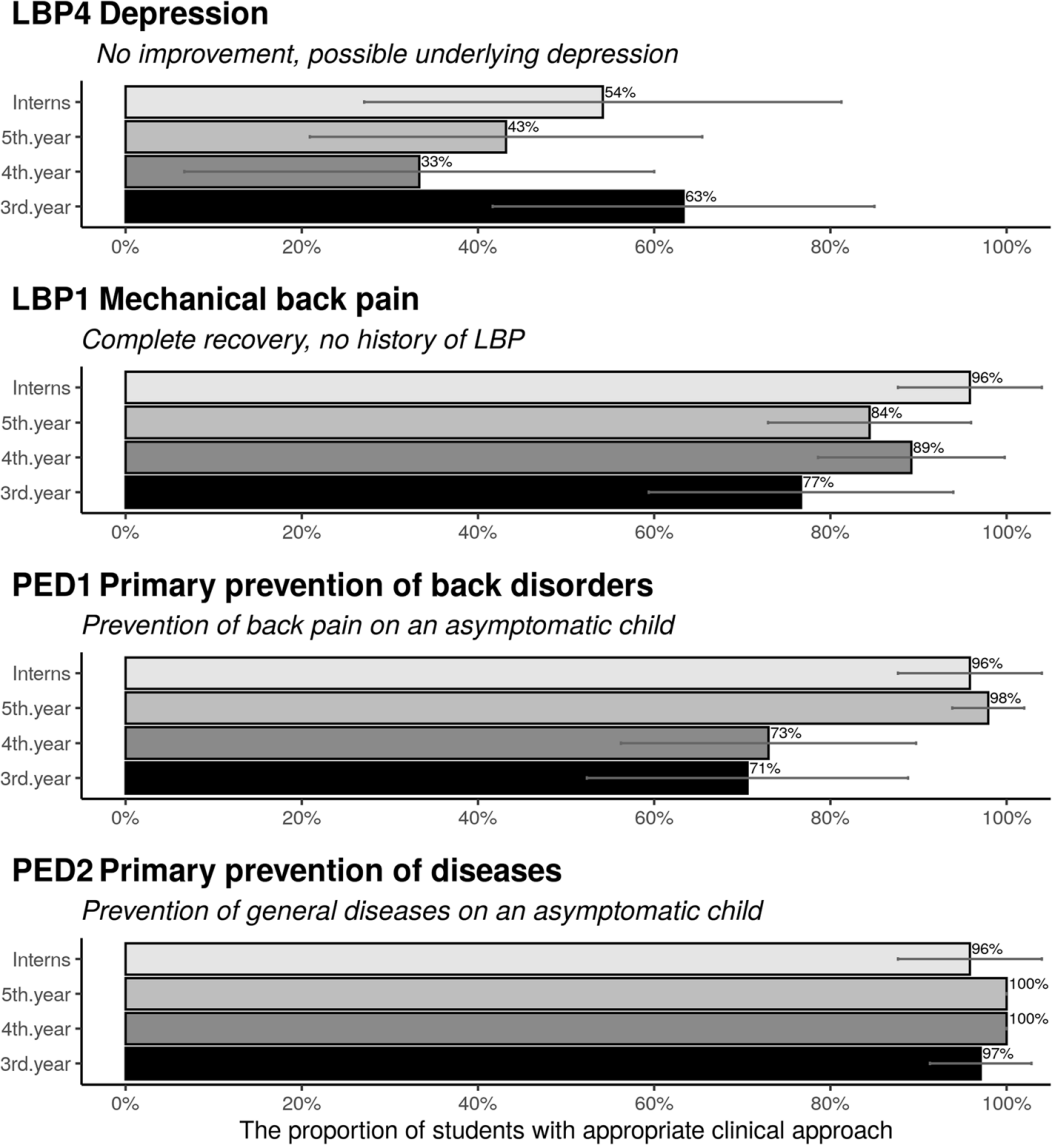
Proportion of Danish chiropractic students and postgraduate interns who were able to select non-indications for spinal manipulation. (Error bars represent 95% confidence intervals (n = 146))

The ability to identify non-indications is depicted in Fig. 2. Similarly, to the case with the pain aggravation, only between 33 and 63% would refer a depressive patient for a second opinion, with no significant differences between the years of study, again with wide confidence intervals. The participants answered ‘appropriately’ in 71 to 100% of the instances for the remaining cases.

In the case concerning an asymptomatic patient, between 77% (3rd year students) and 96% (postgraduate interns) would consider the treatment as completed. Between 71 and 100% would not offer treatment as prevention of spinal pain syndromes or disease in general, for children. Overall, the appropriate answers appear to improve as the academic year of study increases.

#### Ability to determine indications

**Fig. 3:**
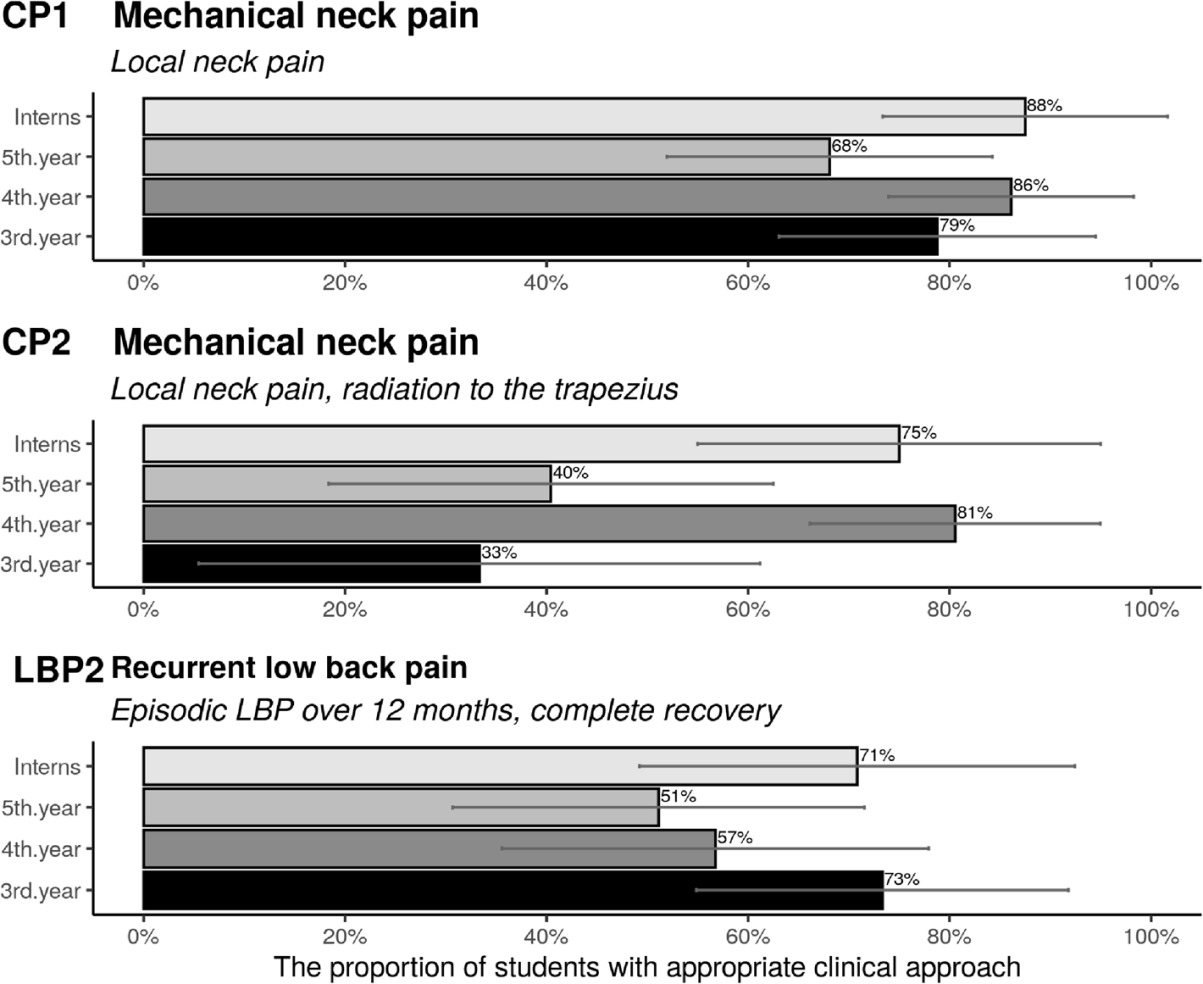
Proportion of Danish chiropractic students’ and postgraduate interns’ ability to select indications for spinal manipulation. (Error bars represent 95% confidence intervals (n = 146))

Figure 3 shows the participants’ ability to identify ‘appropriate’ chiropractic clinical indications. In general, the participants found it challenging to identify indications for treatment in the following situations: i) a case describing simple mechanical neck pain, where only between 68 and 88% would treat this on their own, ii) when the pain radiates to the shoulder, the frequency drops to between 33 and 81% with large differences observed for the 3rd and 5th years vs. 4th year and postgraduate interns, and iii) a potential maintenance care patient would be given a maintenance care treatment plan by only 51 to 73% of participants.

To conclude, participants were most likely to treat simple, local neck pain on their own, less able at identifying a maintenance care patient, and less interested in providing treatment on their own for simple neck pain with pain radiating to the shoulder, with some between-class estimate differences.

#### Clinical approach by academic year and sex

In general, there was a tendency that 5th year students and interns answered more clinically ‘appropriately’, but this was not statistically significant. Sex had no impact on the outcome of the regression analyses.

#### Associations between students’ conservative score and their ability to make ‘appropriate’ clinical decisions

It was not possible to perform the logistic regression for the conservatism groups testing for clinical decisions using the four scores, as only three students were identified as highly conservative (group 4). Thus, these students were omitted from the initial analysis and described individually. The unadjusted and adjusted (sex and academic year of study) analyses for all other participants are presented in Tables 6 and 7. Both the unadjusted and the adjusted associations between conservatism group and an inability to determine non-indications showed that the most conservative group (i.e. group 3) found this most difficult. However, none of the differences between groups 1 and 3 were statistically significant.

**Table 6:** Unadjusted associations between level of chiropractic conservatism in chiropractic students and their inability to determine contra-indications, non-indications, or indications to chiropractic treatment (*N* = 146)

	Contra-indications		Non-indications				Indications		
Conservatism group	CP3, OR[95%CI]	LBP3, OR[95%CI]	LBP4, OR[95%CI]	LBP1, OR[95%CI]	PED1, OR[95%CI]	PED2, OR[95%CI]	CP1, OR[95%CI]	CP2, OR[95%CI]	LBP2, OR[95%CI]
*1 (index)*	1	1	1	1	1	1	1	1	1
*2*	2.18 (0.68–7.65)	2.11 (0.95–4.83)	1.26 (0.60–2.67)	2.16 (0.67–7.57)	1.53 (0.51–4.68)	Too few cases	1.07 (0.44–2.58)	1.58 (0.76–3.32)	0.46 (0.21–0.99)
*3*	1.6 (0.21–8.26)	1.59 (0.51–5.55)	1.72 (0.57–5.62)	3.87 (0.85–16.83)	3.51 (0.91–12.99)	Too few cases	0.48 (0.07–1.97)	1.02 (0.33–3.01)	0.50 (0.14–1.55)

OR = Odds ratio. 95%CI = 95% Confidence intervals. CP3 = Cervical pain and signs of upper motor lesion. LBP3 = Low back pain aggravation after treatment. LBP4 = Low back pain with underlying depression. LBP1 = Mechanical Low back pain. PED1 = Primary prevention of back disorders. PED2 = Primary prevention of diseases . CP1 = Mechanical neck pain. CP2 = Mechanical neck pain with radiation to the trapezius. LBP2 = Recurrent low back pain

**Table 7:** Adjusted associations between level of chiropractic conservatism in chiropractic students and their inability to determine contra-indications, non-indications, or indications to chiropractic treatment (N = 146)

	Contra-indications		Non-indications				Indications		
Conservatism group	CP3, OR[95%CI]	LBP3, OR[95%CI]	LBP4, OR[95%CI]	LBP1, OR[95%CI]	PED1, OR[95%CI]	PED2, OR[95%CI]	CP1, OR[95%CI]	CP2, OR[95%CI]	LBP2, OR[95%CI]
*1 (index)*	1	1	1	1	1	1	1	1	1
*2*	2.33 (0.63–9.15)	1.79 (0.75–4.41)	1.52 (0.67–3.56)	2.82 (0.81–10.83)	1.77 (0.50–6.66)	Too few cases	1.51 (0.55–4.20)	1.94 (0.80–4.91)	0.58 (0.25–1.34)
*3*	2.25 (0.27–14.44)	1.23 (0.36–4.67)	2.05 (0.61–7.44)	4.83 (0.93–25.18)	2.66 (0.57–12.86)	Too few cases	0.83 (0.11–4.07)	1.14 (0.30–4.23)	0.61 (0.16–2.06)

OR = Odds ratio. 95%CI = 95% Confidence intervals. CP3 = Cervical pain and signs of upper motor lesion. LBP3 = Low back pain aggravation after treatment. LBP4 = Low back pain with underlying depression. LBP1 = Mechanical Low back pain. PED1 = Primary prevention of back disorders. PED2 = Primary prevention of diseases . CP1 = Mechanical neck pain. CP2 = Mechanical neck pain with radiation to the trapezius. LBP2 = Recurrent low back pain

#### Group 4 (the most conservative students)

Three students were identified as belonging to the most conservative group (group 4). All were females with a conservatism score of 8 or 9 out of 10. As one of these students stopped answering after the cervical cases, the answers regarding the LBP case are missing. Two out of the three students answered inappropriately on the two final cervical cases, including not finding an upper motor lesion unsuitable for treatment. Also, the two students with available responses answered inappropriately on the simple mechanical back pain case and the case of pain aggravation. In both instances, more treatment would be provided instead of treatment termination. Student number three would treat primary prevention of both spinal pain and general diseases, which was abnormal behavior compared to the rest of the cohort. For a detailed description of each student see Table 8.

**Table 8:** A detailed overview of the three chiropractic students in the highest conservatism group

Students in conservatism group 4	Case #1	Case #2	Case #3
*Demographics*	Female 3rd year conservatism score of 8	Female 4th year conservatism score of 8	Female 3rd year conservatism score of 9
*CP1 – Mechanical neck pain*	**I would treat the patient with the assistance of another paramedic**	I would treat the patient on my own	I would treat the patient on my own
*CP2 – Mechanical neck pain with radiation*	**I would treat the patient whilst asking the opinion of a specialist**	I would treat the patient on my own	**I would treat the patient with the assistance of another paramedic**
*CP3 – Upper motor lesion*	I would not treat the patient but refer him out	**I would treat the patient with the assistance of a general practitioner**	**I would treat the patient with the assistance of a general practitioner**
*PED1 – Primary prevention of back disorders*	Don’t know	Probably not	**Probably yes**
*PED2 – Primary prevention of diseases*	Probably not	Don’t know	**Probably yes**
*LBP1 – Mechanical back pain*	No reply	**I would try a few more treatments and perhaps change my treatment strategy, until I am sure that I cannot do any more.**	**I would follow this patient for a while, attempting to prolong the time period between visits until either the patient is asymptomatic or until we have found a suitable time lapse between check-ups to keep the patient symptom-free.**
*LBP2 – Recurrent low back pain*	No reply	I would follow this patient for a while, attempting to prolong the time period between visits until either the patient is asymptomatic or until we have found a suitable time lapse between check-ups to keep the patient symptom-free.	**I would advise the patient to seek additional treatment whilst following the patient.**
*LBP3 – Pain aggravation*	No reply	**I would try a few more treatments and perhaps change my treatment strategy, until I am sure that I cannot do any more.**	**I would try a few more treatments and perhaps change my treatment strategy, until I am sure that I cannot do any more.**
*LBP4 – Depression*	No reply	**I would follow this patient for a while, attempting to prolong the time period between visits until either the patient is asymptomatic or until we have found a suitable time lapse between check-ups to keep the patient symptom-free.**	I would refer the patient to another health care practitioner for a second opinion.

Bold indicates an ‘inappropriate’ answer

## Discussion

### Summary of the results

#### Chiropractic conservatism

This survey shows that chiropractic students attending an undergraduate institution in close collaboration with a medical faculty generally do not subscribe to the conservative chiropractic concepts. To our surprise, however, they have picked up some other tenets that are not part of their curriculum. Approximately 20% answered ‘inappropriately’ about the connection between spinal manipulation and its ability to intervene with the nervous system in instances such as *adjustments can: prevent diseases in general and help the immune system.* Some also believe that a*djustments can: prevent degeneration of the spine and improve the health of infants.* However, this pattern improved with academic year of study and was limited to only three students placed in the highest conservatism group (group 4).

#### Clinical appropriateness

The included chiropractic students were generally able to identify non-indications and contra-indications to spinal manipulation, albeit some did not recognize that a patient with only minor improvement and a possible underlying depression should be referred out. Mostly, the students also appropriately identified an upper motor lesion case as unsuitable for treatment but were less inclined to suggest that a patient who gradually gets worse should be referred out and should also be considered a potential contra-indication for treatment. As for indications, they would treat a simple mechanical neck pain case on their own but were more reluctant to do so when the pain radiated towards the shoulder. No statistically significant changes appeared when controlling for sex and academic year of study.

#### Chiropractic conservatism and clinical appropriateness

While the level of conservatism was not statistically significantly associated with the ability to determine appropriate clinical decisions, we observed a tendency that the higher conservative groups had difficulty regarding the management of non-indicated cases. In general, the clinical appropriateness appears to improve with increasing academic year of study. The three students (2%) who belonged to the highest conservative group (group 4) were treated as outliers, and their replies should raise cause for concern, as patient safety was jeopardized in at least two of the answers they provided. These findings indicate that approximately a third of the students in the Danish course adhere to at least some of the original chiropractic ‘philosophy’ [3].

### Comparison with previous surveys

**Fig. 4:**
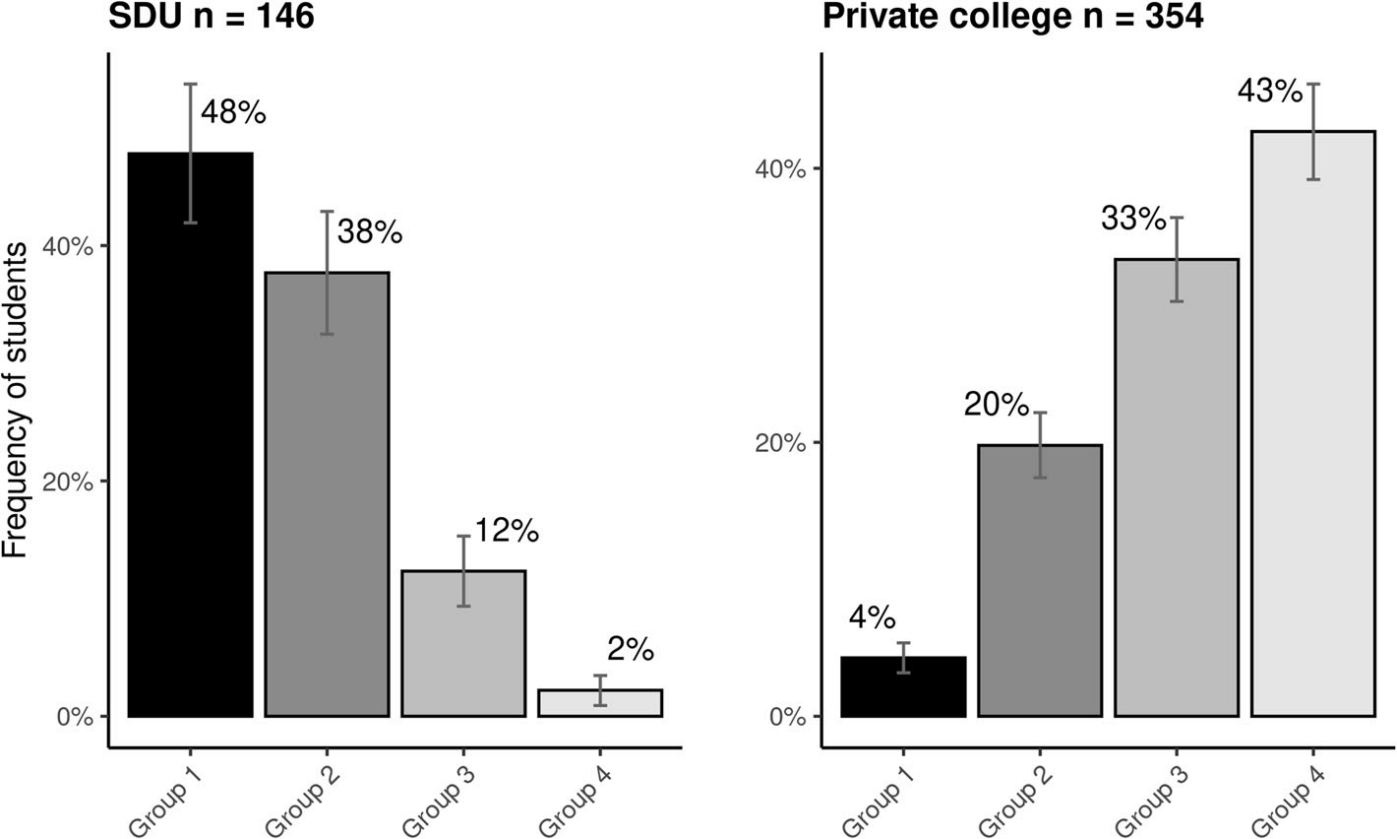
Difference in levels of chiropractic conservatism among students in a chiropractic course placed in a university and closely integrated with a medical faculty (left) compared to students attending a private chiropractic college (right). (Group 1 indicates low conservatism, whereas group 4 indicates highly conservative thinking, SDU = University of Southern Denmark)

The chiropractic conservatism profile in this study was quite similar to that of the students from two other university-based courses in Australia [8]. Both have inappropriate tendencies on the ability of spinal adjustments to improve the health of infants and help the body function at 100% of its capacity. However, this cohort was very different from that reported in the study of the European non-university based course [9]. In fact, there was a remarkable inverse pattern for the four groups, as the majority of the Danish students were placed in the two lowest conservatism groups while the majority of the private college students were in the two highest groups (Fig. 4).

The Danish students’ clinical choices were similar to those reported in the previous studies from the private chiropractic college [9] and the university-based institutions in Australia [13]. With all three cohorts scoring appropriately on the upper motor lesion case. The private college cohort scored more appropriately than the two University cohorts on the case of pain aggravation. Concerning the indications, an overall agreement was found. However, the two university cohorts struggled in identifying the maintenance care patient compared to the private college. Comparable to this cohort, the level of conservatism at the private college did not significantly modify the responses for the indicated cases [9].

Discrepancies between the conservative and non-conservative students regarding the management of non-indicated cases were found in the Danish study and the private college [9], whereas this association was not tested in the Australian study. In the Danish university student cohort, which was not very conservative, these results never reached statistical significance. Contrary, in the very conservative private school, exceptionally strong associations were observed between a firm adherence to the chiropractic conservative belief system and the willingness to treat non-indicated cases. For example, in that course, students in the highest category of conservatism were 20 times more likely than those in the lowest category to offer children treatment to *prevent diseases from developing* [9]. This suggests that the Danish students’ level of conservatism had a lesser impact on their clinical appropriateness than for private college students. While the two state universities [13] did not provide conservatism scores or included the cases with preventive treatment for a 5-year old, their scores concerning the patient with depression were comparable to this and the private college study. In contrast to the other cohorts [9, 13], the Danish students had no issue terminating treatment for a simple mechanical low back patient.

### Potential reasons for chiropractic conservatism at a university program closely collaborating with a medical faculty

During their years of study, students are exposed to many different and exciting chiropractic concepts. Vitalism and its inherent promise of helping many varied and challenging conditions, despite the state of current scientific evidence suggesting otherwise, would clearly appeal to some students’ altruistic nature. However, the purpose of chiropractic education should include providing students with a scientifically acceptable and clinically realistic view of their future profession. This does indeed appear to be the case in the Danish course, where scientific evidence and clinical plausibility becomes more evident in the latter academic years, whereas this was not shown in the other state university setting [8], and was not reported in the private chiropractic college study [9]. Arguably, this highlights the potential value of close collaboration with a medical faculty.

Nonetheless, quite a few Danish chiropractic students still hold at least some unusual beliefs that would surprise the university faculty and, most likely, the Danish health authorities. This was unexpected, when considering the educational setting of these students, which includes a curriculum that does not contain conservative chiropractic ideas, and where concepts like spinal ‘subluxations’ are taught exclusively as objects of historical interest. Furthermore, both the course management and student organization are signatories of the *International Clinical and Professional Chiropractic Education Position Statement* [26].

Whether this chiropractic conservatism among students results from factors acting within the institutions [27] or are concepts picked up from outside is difficult to say. This might also partly be an intrinsic problem, inherent in the student body. Altruistic students with an acceptance of alternative treatment approaches may well become attracted to an alternative to the scientific approach in the information material of an educational institution, and they could become accepted into the pre-graduate course, particularly if there is a need for large student intake, as there is in private colleges. However, at the Danish university, a conscious effort is exerted in informing applicants on the musculoskeletal and scientific focus of the education [28]. Nevertheless, although admittance procedures for the education at the Danish course have been tightened in recent years, the selection process of students could be inadequate.

The degree of conservatism in the surrounding chiropractic profession, as well as the type of non-scientific courses offered by non-university educators, which students attend in their free time, may also play a role. Indeed, we noted a greater difference in conservatism between 3rd year students and 5th year students than between 3rd year students and postgraduate interns. This could reflect the introduction of new, external influences on the interns, who during the internship, work with other practicing chiropractors and only return to the university for periodic meetings. If website content can be taken as a measure of conservatism, the prevalence of conservative chiropractic ideas among practicing chiropractors in Denmark is surprisingly higher than anticipated [29], and this could exert an unduly influence on clinical interns. It is also possible that students pick up conservative chiropractic convictions and ideas through social media [30]. Finally, external lecturers working within the university may also include non-evidence-based concepts and views in their teaching of students, thus bypassing the university methods of “vetting” new lecturers. Most of these arguments were also raised regarding the two other student cohorts [8, 9, 13] as potential causes for the non-evidence-based reporting.

The sine qua non of clinical chiropractic is spinal manipulation, which plays a central role in the often tricky process of adopting an identity as a chiropractor. Ascribing wider effects to spinal manipulation than that allowed by the evidence and with a mechanistic approach could potentially help smooth such a transition for some students. It could even be argued that because Danish chiropractic students spend the majority of their time in class together with their medical counterparts, who later go on to work in specialties ranging from child-psychiatry to forensic pathology, a chiropractic scope of practice limited to musculoskeletal pain could be restrictively narrow for some. This, in turn, could open the gate for charismatic conservative chiropractic lecturers and opinion-makers, who offer a gold-trimmed chiropractic identity rooted in spinal manipulation as a panacea. Regardless of the reasons, it appears necessary that the course work given on the indications for spinal manipulation, in particular, has to be extended from simple contra-indications to include lectures on when it should be applied and, more importantly, when it should *not* be applied.

### The impact of chiropractic conservatism on appropriate clinical decision making

In our study, the association between conservatism and the ability to make appropriate clinical judgments did not reach statistical significance, possibly due to the low number of highly conservative students. On first look, our data, therefore, suggest that the impact of conservatism on critical clinical judgments (non-indications) was limited. Nevertheless, just as in the previous survey [9], those with the highest conservatism score were shown to have a higher unsuitable clinical decision pattern. The authors of the private college study argue that these findings fit the profile of the original philosophical subluxation-based model where “everything” is treatable [9]. The authors of the study on the students from the state universities, who also had difficulty identifying non-indications, suggests that this could be due to the students having a “try it and see how it goes” approach, were overconfident, or utterly lacked knowledge regarding when not to administer treatment [13]. Overall, the students in our cohort had no substantial issues with non-indications, except for the case concerning a depressed patient. While the other arguments are plausible, due to the integral part that Danish chiropractors play in the healthcare system, we would add that the Danish students see themselves as becoming gatekeepers and would rather collaborate on a solution for the patient as opposed to merely terminating the treatment. This could account for their reluctance to terminate treatment for the patient with underlying depression and the patient experiencing pain aggravation.

### Recommendations

In what would arguably be a ‘flagship’ for a modern chiropractic university program, the presence of some’ conservative’ views clearly shows that conservative chiropractic concepts need to be dealt with up-front and transparently with the consequences it may have on patients foremost in mind. Therefore, the sources of influence that give rise to the adherence to such viewpoints must be identified and dealt with accordingly to ensure that young students become safe and ethical practitioners.

### Methodological considerations

An obvious strength of the current study is the ability to compare our findings with those of other studies [8, 9, 13]. Nevertheless, a full comparison of all items was not possible, as the data reporting was not identical across studies.

There are some potential weak points to consider regarding this study. It is possible that the opinions of non-responders could have altered the results, but as this was an anonymous survey, it is not possible to conduct a responder/non-responder analysis. The response rates per year of study ranged from acceptable to good, and we have no reason to suspect that the students who either were absent on the day of invitation or were uninterested in responding would have a remarkably different profile that could substantially change the results. However, 8 students chose to terminate their survey in the initial section for reasons unknown. We speculate that these students did not understand the questions or disliked responding to the questions. However, judging by their intermediate score, they did not appear to be highly chiropractically conservative.

The clinical cases in the questionnaire, which had already been administered in two other student settings [9, 13], was translated to Danish in an appropriate fashion, and piloted before use. However, some uncertainty may have arisen due to the lack of clinically oriented detail. In general, it is possible that the items used in this and previous questionnaires [9, 13] had poor content validity [31] despite having gone through pilot-tests. For instance, the question a*djustments can improve the health of infants*, might have been interpreted to specifically refer to infantile colic, a condition very commonly treated by chiropractors in Denmark [32]. Thus, answers may reflect a conviction that ‘adjustments’ improve the health of infants in specific circumstances, and not as a general effect. This is speculative, of course, and would require further research to clarify. However, the fact that the score of conservatism (0–10), when tested in one of these studies [9], corresponded in such a logical manner with the inability to respect a number of chiropractic non-indications, indicates that the ten items, on the whole, validly capture individuals who accept the chiropractic conservative concepts.

Lastly, and related to content validity, while the terms ‘subluxations’ and ‘adjustments’ are not typically included in the Danish chiropractic curriculum, we assume that the students would have picked up these terms from their international reading material, and the pilot-tests did not reveal any such problems.

Therefore, we suggest a qualitative follow-up study to investigate i) the validity of items and ii) what sources of influence gave rise to the approximately 20% who answered ‘inappropriately’ on some undoubtedly antiquated viewpoints.

As our data were collected cross-sectionally, our suggestion of the differences between 3rd, 4th, 5th years students and postgraduate interns is not necessarily indicative of development over time. Obviously, only a longitudinal study can establish whether students change over time.

Another potential limitation is that we compared the results only to other chiropractic institutions and not with a similar healthcare profession. Perhaps a fraction of medical students also believe in nineteenth century medical concepts such as smoking is good for you, and ‘miasmas’ are the cause of cholera, and other theories that became obsolete around the same time as chiropractic conservatism emerged [33–35].

## Conclusion

Contrary to previous studies on chiropractic educational institutions, students in a university-based chiropractic program, in close collaboration with a medical faculty, expressed only some notions of chiropractic conservatism, most notably regarding the purported effects of spinal ‘adjustments’/ manipulation rather than the ill effects of spinal ‘subluxation’ / spinal dysfunctions.

Similarly to the previous studies, these students displayed reasonable attitudes towards cases that are clinically appropriate for them to treat. However, contrary to students in a private chiropractic school, the degree of conservatism was not significantly associated with clinical appropriateness, as only a weak and non-significant tendency was observed in the non-indicated cases. i.e. the students with higher conservatism scores had more difficulty identifying non-indications to spinal manipulations.

Thus, chiropractic students enrolled in a university-based education in a medical environment are not immune against chiropractic conservatism, but such education does appear to attenuate chiropractic conservatism and its clinical consequences. While some surprising tendencies occurred, we consider our hypotheses as confirmed.

## Supplementary Information

**Additional file 1.**

**Additional file 2.**

**Additional file 3.**

## Data Availability

The data are available in a fully anonymized format from the corresponding author upon reasonable request.

## Abbreviations

SDU: University of Southern Denmark
OR: Odds ratio
