# Supplementary material for: Chiropractic conservatism among chiropractic students in Denmark: prevalence and consequences
Source: Chiropr Man Therap. 2020 Dec 4;28:64. doi: 10.1186/s12998-020-00352-3 (PMC7716499; doi:10.1186/s12998-020-00352-3)
Supplement: Supplementary file 2 — Additional file 2. [file 12998_2020_352_MOESM2_ESM.docx]

### Supplementary file 2

### Anonymous survey on chiropractic students

**Title of the project:** An investigation of the beliefs and clinical acumen for the chiropractic students at the University of Southern Denmark

**Contact:** Casper Glissmann Nim (casper.nim@rsyd.dk)

**Location:** University of Southern Denmark

**Aim:** To describe i) the proportion of students that follow the principles of chiropractic conservatism ii) if their clinical assessment is suitable iii) and if this is associated with the degree of conservatism.

**What is expected of you:** If you wish to be included you have to answer voluntarily and stay anonymous. The survey consists of different clinical cases and 10 questions regarding subluxations (spinal dysfunctions) and spinal manipulation. The answering time is approximately 10 minutes.

**Concept to be included, and your right to withdraw your consensus:** Your participation is voluntary and anonymous. If you wish to participate or not has no influence on your grades, status or future relations with the University. If you answer the survey it is assumed that you agree to (i) participation and (ii) publication of your data. As the survey is anonymous, it is impossible for you to edit or delete the information after you have submitted the results of the survey.

**Your right to** **confidentiality and privacy:** The data will be treated as confidential and it will be impossible to recognize students from their answers.

**Benefits about the survey:** The investigation can help us to improve the key factors that influence students’ clinical acumen. Thus, helping the university to identify these students.

**Potential risks:** Participation brings no risk for harm or malaise

**Publication:** The results from this survey will be submitted as a peer-reviewed publication and presented at international scientific conferences.
