# Supplementary material for: Chiropractic conservatism among chiropractic students in Denmark: prevalence and consequences
Source: Chiropr Man Therap. 2020 Dec 4;28:64. doi: 10.1186/s12998-020-00352-3 (PMC7716499; doi:10.1186/s12998-020-00352-3)
Supplement: Supplementary file 3 — Additional file 3. [file 12998_2020_352_MOESM3_ESM.docx]

## Supplementary File 3

**Rationale for ‘appropriate answers’ to clinical cases given to chiropractic students from SDU**

- **Contra-indicated cases**

Neck pain scenario 5

This is the case of an aggravation of a patient who was treated for simple cervical neck pain, returning 23 days after the first consultation. There are now signs of central nervous damages (i.e. hyperreflexia, positive sign if Babinski), and a worsening of the symptoms after four chiropractic treatments. The appropriate choice here is to refer the patient out without treating him because further treatment may aggravate the condition even more.

LBP scenario 8

This is the case of a patient who consulted for low back pain. After six visits, the symptoms were gradually worsening. Even if there was a slight improvement after the second visit (20%), the chiropractic treatment did definitely not improve the patient’s condition. The appropriate choice is therefore to refer the patient out to another health care practitioner for a second opinion/more relevant treatment. This patient may well have a condition that could be worse with chiropractic treatment or perhaps the patient is indeed already getting worse *because* of the treatment.

- **Non-indicated cases**

Primary prevention of back disorders

This is the case of an asymptomatic 5-yr old child who never had back pain. Although the idea may appear logical to some, to our knowledge, there is no evidence which supports the concept that spinal manipulations can prevent the onset of back disorders. The appropriate answer is therefore not to accept this patient as this type of treatment is *non-indicated*.

Primary prevention of diseases

This is the case of a 5-yr old asymptomatic child whose mother asks the chiropractor to prevent the onset of diseases in general through chiropractic treatment. To our knowledge, there is no evidence which supports the concept that spinal manipulations can prevent diseases in general [1]. The right attitude here is therefore not to accept this patient for this type of treatment, which is *non-indicated*.

LBP scenario 1

A short episode of LBP was successfully treated in two chiropractic consultations. There was no previous history of back pain, so it is unlikely that this condition will tend to be recurrent. Also, it looks like the patient is a competent and independent person. The right attitude here is therefore to be available for any new episode if it occurs. It is *not indicated* to continue treatment of this asymptomatic patient for the moment.

LBP scenario 9

The chiropractic treatment has not influenced the course of LBP in this patient. Moreover, the psycho-social profile should be taken into account in this patient who appears tired and moody, indicating a case of depression. It is therefore a *non-indicated* case and the chiropractor should refer this patient to another health care practitioner for a second opinion/more relevant treatment. In other words, there has been no improvement, which becomes a *non-indication* after a reasonable amount of time and chiropractors are not competent to diagnose and/or treat depression.

- **Indicated cases**

Neck pain scenario 1

This is a simple case of a local pain with neither irradiation nor neuro-vascular symptoms in the arm. It is an *indication* to chiropractic treatment.

Neck pain scenario 2

Five days later, the symptoms were slightly worse (pain irradiation into the right shoulder, which could be due to soreness of the Trapezius). The clinical signs were unchanged. It is still an *indication* to chiropractic treatment.

LBP scenario 4

This short episode of LBP was successfully treated in two weeks of treatment. This is a chronic patient who has had several episodes in the past year. Therefore, it is likely that this condition will be recurrent [2, 3] Maintenance care treatments (symptoms-guided or, possibly, clinical findings-guided) are therefore *indicated* for this patient [4, 5].

**REFERENCES**

1. Goncalves G, Le Scanff C, Leboeuf-Yde C: **Effect of chiropractic treatment on primary or early secondary prevention: a systematic review with a pedagogic approach**. *Chiropr Man Therap* 2018, **26**:10.

2. Hestbaek L, Leboeuf-Yde C, Engberg M, Lauritzen T, Bruun NH, Manniche C: **The course of low back pain in a general population. results from a 5-year prospective study**. *Journal of Manipulative and Physiological Therapeutics* 2003, **26**(4):213-219.

3. Lemeunier N, Leboeuf-Yde C, Kjaer P, Gagey O: **Stability of low back pain reporting over 8 years in a general population aged 40/41 years at base-line: data from three consecutive cross-sectional surveys**. *BMC Musculoskeletal Disorders* 2013, **14**(270).

4. Andreas E: **Recurrent and persistent low back pain - Course and prevention.** Karolinska Institutet; 2016.

5. Kongsted A, Hestbaek L, Ammendolia C, Côté P, Southerst D, Schneider M, Budgell B, Bombardier C, Hawker G, Raja Rampersaud Y *et al*: **ECU convention 2017 research presentations**. *ECU convention 2017 research presentations.* vol. 25: Chiropractic & Manual Therapies; 2017.
